# The fecal microbiota of wild and captive raptors

**DOI:** 10.1186/s42523-020-00035-7

**Published:** 2020-05-06

**Authors:** Bruno C. M. Oliveira, Maureen Murray, Florina Tseng, Giovanni Widmer

**Affiliations:** 1 Department of Infectious Disease & Global Health, Cummings School of Veterinary Medicine at Tufts University, North Grafton, MA 01536 USA; 2 Universidade Estadual Paulista (UNESP), Faculdade de Medicina Veterinária, Araçatuba, Brazil

**Keywords:** Raptor, Fecal microbiota, Rehabilitation, *Actinobacteria*, 16S amplicon sequencing

## Abstract

**Background:**

The microorganisms populating the gastro-intestinal tract of vertebrates, collectively known as “microbiota”, play an essential role in digestion and are important in regulating the immune response. Whereas the intestinal microbiota in humans and model organisms has been studied for many years, much less is known about the microbiota populating the intestinal tract of wild animals.

**Results:**

The relatively large number of raptors admitted to the Tufts Wildlife Clinic on the Cummings School of Veterinary Medicine at Tufts University campus provided a unique opportunity to investigate the bacterial microbiota in these birds. Opportunistic collection of fecal samples from raptors of 7 different species in the orders *Strigiformes*, *Accipitriformes*, and *Falconiformes* with different medical histories generated a collection of 46 microbiota samples. Based on 16S amplicon sequencing of fecal DNA, large β-diversity values were observed. Many comparisons exceeded weighted UniFrac distances of 0.9. Microbiota diversity did not segregate with the taxonomy of the host; no significant difference between microbiota from *Strigiformes* and from *Accipitriformes/Falconiformes* were observed. In contrast, in a sample of 22 birds admitted for rehabilitation, a significant effect of captivity was found. The change in microbiota profile was driven by an expansion of the proportion of *Actinobacteria*. Based on a small number of raptors treated with anti-microbials, no significant effect of these treatments on microbiota α-diversity was observed.

**Conclusions:**

The concept of “meta-organism conservation”, i.e., conservation efforts focused on the host and its intestinal microbiome has recently been proposed. The observed effect of captivity on the fecal microbiota is relevant to understanding the response of wildlife to captivity and optimizing wildlife rehabilitation and conservation efforts.

## Background

The intestinal microbiome is being increasingly recognized as playing a crucial role in the health of humans and animals, impacting nutrient absorption, immune function and other physiological processes [1, 2]. In wildlife species, microbiome characterization and monitoring is emerging as a tool in conservation, particularly for captive breeding and in the management of endangered species. Notable examples are studies of the microbiota of the critically endangered Attwater’s prairie chicken and the kakapo, an intensively managed flightless New Zealand parrot [3–6]. Knowledge derived from the analysis of the microbiota is also being applied to amphibians [6]. While studies on the intestinal microbiome of wildlife are increasing in number, a recent literature review found that greater than 90% of these studies focus on mammals [7]. Studies on birds are concentrated on a small number of avian taxa [1]. With the exclusion of vultures [8], to our knowledge the intestinal microbiome of carnivorous birds has not been explored using next-generation sequencing. The oral microbiota of an urban population of Cooper’s hawks was also investigated [9]. The focus of this research was to assess the association between the hawks’ age and oral pH to explain the age-dependent prevalence of *Trichomonas gallinae* in these birds and, therefore, the fecal microbiota was not sampled. A recent review of research on the avian gut microbiome [2] reveals a strong bias towards commercially important birds, like chicken and turkey, or evolutionary unique species, like the leaf-eating hoatzin [10, 11] and penguins [12].

Small sample sizes, opportunistic sampling, uncontrolled environmental variables (e.g., diet, age, gender, geographical origin, season) are typical limitations of surveys of wildlife microbiota. Research on the effect of captivity on the intestinal microbiota has compared wild and captive conspecific animals. An example of such a study compared the fecal microbiota of wild and matching captive species housed in eight zoos [13]. As no species was represented by more than 10 captive or wild individuals, the absence of an observed effect of captivity may reflect the small sample size and the large number of uncontrolled variables. Two studies on avian species have revealed changes in the composition of bacterial populations due to captivity when comparing wild and captive birds [14, 15].

The need to broaden the study of host-associated microbiota to a wider selection of taxa has been noted [7]. To validate the importance of the meta-organism (host and microbiome) in species management and re-introduction, baseline information on the healthy microbiota of a diverse set of species is needed. With respect to the management of bird species, the effect of captivity and treatment with antimicrobials and antiparasitics on the microbiota of birds with different gastro-intestinal (GI) physiology remains unexplored. In humans and rodents, the effect of such treatments is relatively well understood [16–18]. It is unknown to what extent this knowledge can be extrapolated to other species particularly to species like raptors which consume a very different diet. This topic deserves attention as antibiotics and antiparasitics are commonly administered to birds in rehabilitation.

Whereas hawks and owls are commonly referred to as birds of prey, the GI physiology of these two groups of birds is not identical. The proventriculus of *Accipitriformes/Falconiformes* (hawks, eagles, and falcons) is significantly more acidic (pH 1.6) than that of *Strigiformes* (owls; pH 2.35) [19]. Hawks fully digest the bones of their prey, whereas owls do so only partially [20]. It has been hypothesized that stomach acidity is an adaptation to diet; birds that feed on carrion or have a predatory life style are thought to be protected from foreign microorganism and bacterial toxins by extremely low stomach pH [21]. *Accipitriformes/Falconiformes* and *Strigiformes* also show anatomic differences with the former having vestigial ceca and the latter having enlarged ceca, a feature more commonly seen in herbivores [20].

The main goal of this study was to characterize the fecal microbiota of different species within three orders of raptors and to determine potential effects of medical therapy and captivity. Ethical considerations precluded experimental interventions to assess the response of the microbiota to dietary or other perturbations.

## Results

### Global analysis of raptor microbiota

**Fig. 1:**
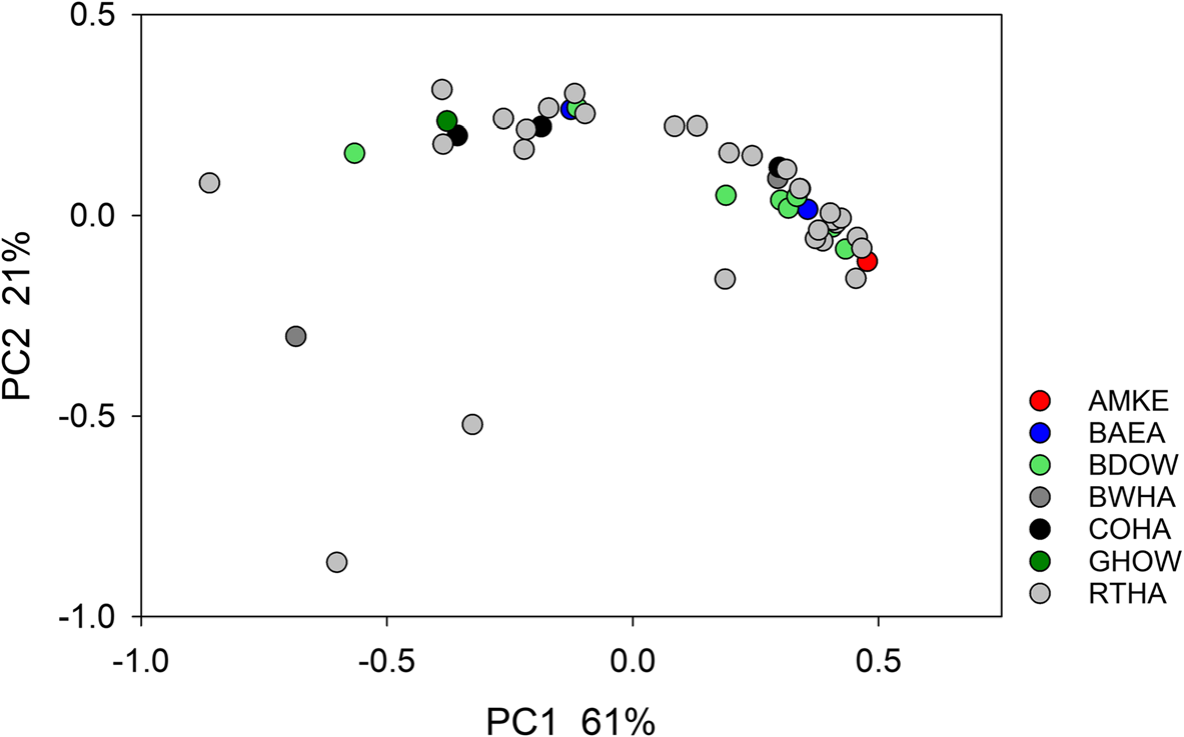
Principal Coordinate Analysis of fecal microbiota of 46 raptors based on EMD. Color indicates species; green colors are owls

The bacterial microbiota profile collected from 46 raptors was highly diverse. This observation is illustrated using weighted UniFrac distance. This metric ranges in magnitude from 0 for identical populations to 1 for populations that share no sequences [22]. The mean distance between the 46 raptor microbiota was 0.78 (*n* = 1035, SD = 0.20). A total of 459 (44%) of distance values were larger than 0.900 (Fig. S1). Given the large proportion of near-maximal UniFrac values, EMD was used in all subsequent analyses of β-diversity. Mean EMD for the 1035 pairwise comparisons between 46 microbiota was 0.527 (SD = 0.302). Consistent with the PCoA plot shown in Fig. 1, 16S sequences from *Accipitridae* (*n* = 37) and *Strigidae* (*n* = 9) were not significantly clustered (ANOSIM R = − 0.045, *p* = 0.639).

The taxonomic classification of 214,464 curated 16S sequences (Table S1) revealed that *Firmicutes* is the most abundant phylum-level classification, representing 60.0% of all sequences. With 24.8%, Proteobacteria were about half as abundant, followed by *Actinobacteria* (6.9%). In average, *Bacteroidetes* represented only 4.2% of all sequences. The only other phylum that exceeded 1% abundance was *Fusobacteria* (1.7%).

### Died/euthanized vs. survivors

A total of 22 of the 46 birds were determined to be good candidates for treatment and rehabilitation, whereas 24 were euthanized or died shortly after admission due to the severity of their injury or illness. Among the 22 birds that were deemed candidates for rehabilitation, 7 species were represented, including 2 species of owls and 3 species of hawks, an American kestrel and a bald eagle. Reflecting the heterogeneous nature of the injuries and conditions from which the birds died or which led to the decision to euthanize them, the fecal microbiota in the birds that did not survive was slightly more heterogeneous. Mean pairwise EMD distance between these birds was 0.56 (*n* = 276, SD = 0.32), which is larger than the mean of 0.50 (*n* = 231, SD = 0.28) between birds admitted for rehabilitation. The difference, however, is statistically not significant (Mann-Whitney Rank Sum Test, *p* = 0.067).

**Fig. 2:**
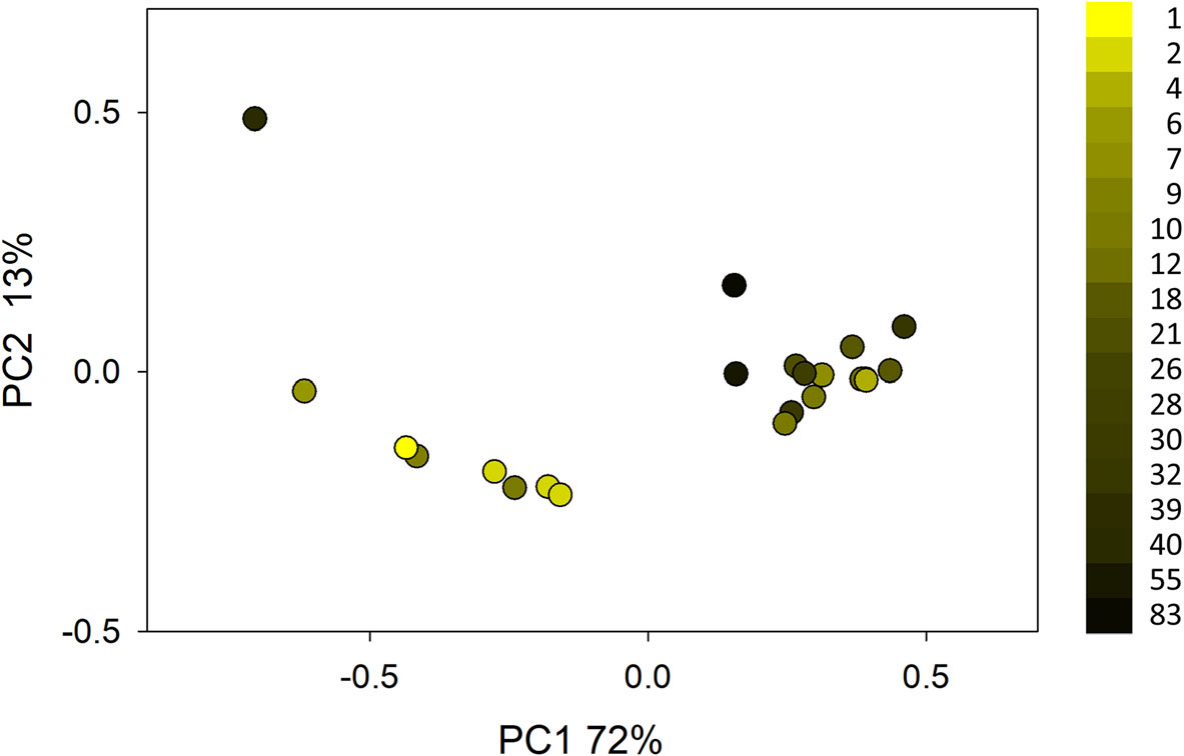
Principal Coordinate Analysis of 16S sequences from 22 fecal microbiota from surviving birds housed at TWC between 1 and 83 days. Samples from 24 of 46 birds that were euthanized because of the severity of their injuries, or which did not survive, were excluded. A sample from a long-term captive BAEA was also excluded. Each dot represents a fecal sample, colored according to duration of stay at the time a fecal sample was collected for analysis. PCoA is based on EMD. The color key indicates days in captivity

A PCoA of sequence data from 22 birds admitted for rehabilitation is shown in Fig. 2. Coloring the data points according to duration of captivity revealed a gradient, suggesting that the microbiota changes with time in captivity, regardless of bird species. CCA was used to test for statistical association between days in captivity and OTU profile. To assess the effect of captivity on the microbiota, the variable “bird species” was excluded by defining it as a covariate. CCA showed that the variable “days in captivity” was significantly associated with the OTU profile (pseudo-F = 1.8, *n* = 22, 271 OTUs, *p* = 0.014).

To investigate the changes in the bacterial community during captivity underlying the CCA results, OTUs were ranked by percent variation explained by “days in captivity”. This percentage value is sometimes abbreviated as *FitE* [23]. The phylum-level taxonomic classification of the 10 OTUs which were best explained by days in captivity (10 highest *FitE* values) were then compared with the taxonomy of entire population of 271 OTUs. This analysis revealed a 4.3-fold larger proportion of *Actinobacteria* OTUs than expected from the classification of the 271 OTUs (Table 1). A Goodness-of-Fit test comparing the observed phylum frequency with the phylum frequency of the 271 OTUs indicates that the observed frequencies were significantly different (Chi-square = 155.2, 5 d. f., *p* < 0.001). With a frequency of 60% (6/10), OTUs classified in the phylum *Actinobacteria* were over-represented in the 10 OTUs which best correlated in abundance with days in captivity (high *FitE*). Five of the 6 high-*FitE* OTUs were classified as *Actinomycetales*.

**Table 1:** Taxonomy of OTUs with best fit to variable “days in captivity”

Phylum	Hi FitE OTUs	Observed frequency	271 OTUs	271 OTUs freq.
unclassified bacteria	1	10	16	6
*Actinobacteria*	6	60	37	14
*Firmicutes*	3	30	135	50
*Proteobacteria*	0	0	48	18
*Bacteroidetes*	0	0	29	11
other	0	0	6	2
sum	10	100	271	101

**Fig. 3:**
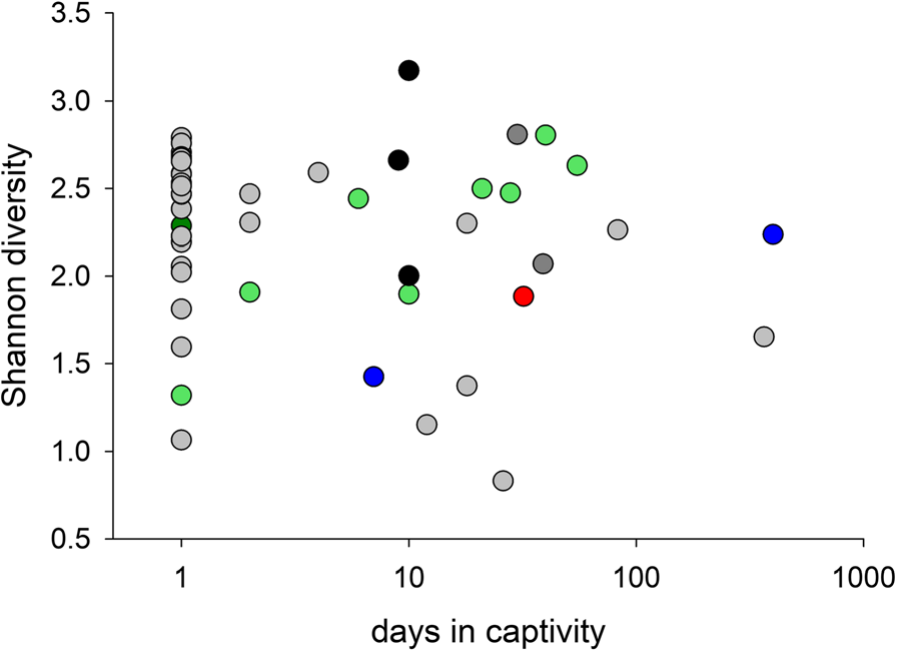
Lin-log plot of microbiota Shannon diversity vs. time in captivity for 46 raptors indicates that captivity does not depress microbiota diversity. Datapoints are colored by species as in Fig. 1. Datapoints at x = 1 represent birds that were euthanized or died within 24 h of admission

In light of the observed changes in the microbiota profile during captivity, we also investigated whether captivity impacted α-diversity. The data did not support our hypothesis that captivity reduces α-diversity (Fig. 3). Using Berger-Parker diversity instead of Shannon gave essentially the same result (Fig. S2).

### No detectable impact of treatment with antibiotic and antifungal

Of the 46 birds included in the study, 3 were treated with 1 to 5 intramuscular injections of the antibiotic ceftiofur crystalline free acid and 1 with the antifungal itraconazole given orally. Samples from these birds were collected after 6 days of treatment (*n* = 2), 2 days after treatment was terminated (*n* = 1) and 5 days after the treatment was terminated (n = 1). We investigated the effect of antibiotic/antifungal treatment on the fecal microbiota of these birds by comparing Shannon α-diversity from treated and untreated birds (Fig. S3). This analysis did not reveal any treatment effect on microbiota α-diversity.

## Discussion

### Global analysis, α-, β-diversity and taxonomy

The fecal microbiota of carnivorous birds has rarely been studied using high-throughput sequencing methods. To our knowledge, this study represents the largest number of raptor microbiota samples analyzed to date. An extensive review of avian microbiome studies published in 2014 does not reference any studies of raptors [24]. The logistical challenges of sampling raptors in the wild are obvious. Studying raptors admitted to Tufts Wildlife Clinic (TWC) bypassed these challenges and enabled us to gain insight into a specialized intestinal ecosystem. The obvious drawback of our sampling method is the high probability that admitted birds have experienced severe trauma, have a debilitating injury or illness and in some cases may have been unable to feed for extensive periods of time. As a result, their intestinal microbiota may have undergone significant changes. Opportunistic sampling of birds from different species and with a diverse history brings significant challenges, like the potential influence of many uncontrolled variables which are too numerous to account for in a collection of 46 samples. For instance, we did not attempt to analyze the impact of geographical origin. The diverse nature of medications (antibiotics, antihelminthics, anti-inflammatories, antifungal and pain medication) given in different combinations and at different timepoints represented a further challenge. The number of birds subjected to the same treatment was too small to support meaningful statistical analyses. The mission of TWC precludes invasive sampling, raising the possibility that fecal swabs may not accurately represent the hindgut microbiota. A comparative study of five types of samples, including from the large intestine and feces, found that in zebra finches the large intestine microbiota closely resembled the fecal microbiota [25].

The most striking features of the sequence data is the large β-diversity between samples. To what extent the medical history of each bird has magnified this diversity is unknown. To put the average weighted UniFrac distance between raptors of 0.78 in perspective, we compared this value to the only dataset we have generated using the same methodology from non-laboratory animals; the fecal microbiota of 97 horses raised on several New England farms [26]. The mean UniFrac distance in this dataset is 0.55, i.e., 70% of the raptors’ average. No other data from wildlife were generated in our laboratory to assess whether high β-diversity is typical of birds living the wild or has been accentuated by injury and disease. Comparing our results with those from other studies of wildlife is problematic as few studies report raw β-diversity values, and because the effect of different laboratory protocols, 16S sequencing protocols and bioinformatics methods is difficult to gauge. In microbial ecology, the EMD is less frequently used than UniFrac, contributing to the difficulty of comparing our results with those from other studies.

Compared to typical mammalian microbiota profiles, the collection of raptor microbiota analyzed here stand out for a low abundance of *Bacteroidetes* (Table S1). Also unusual, compared to typical healthy human fecal microbiota, is the high abundance of *Actinobacteria*. In 27/46 raptors *Actinobacteria* sequences were more abundant than *Bacteroidetes* sequences. Because of this unusual taxonomy, it was important to ensure that our classification method generates accurate results. Based on sequence data obtained from a synthetic bacterial population (Fig. S4), we can exclude that technical artifacts caused by PCR, primers and sequencing have significantly impacted the taxonomy. In fact, classification of sequences originating from the synthetic population shows that our analysis slightly overestimates the abundance of *Bacteroidetes* sequences (8.8% observed vs. 5% expected) and underestimates by a few percentage points the abundance of *Actinobacteria* (7.2% observed vs. 10% expected). The low *Bacteroidetes* to *Firmicutes* ratio observed in our samples (Table S1) appears to be typical of the gut microbiota of carnivores. Low-throughput analysis of 16S sequences amplified from fecal DNA of two captive cheetahs [27] found a similarly low *Bacteroidetes* abundance. A meta-analysis of numerous avian cecal microbiota suggests that high *Firmicutes* proportions as observed in our raptors (mean abundance = 0.60) is not a feature of the avian microbiota [24]. This conclusion is consistent with a survey of intestinal microbiota of tropical birds belonging to 14 orders [28]. Since no raptors were included in this study, the relatively low Firmicutes proportion observed in these birds provides circumstantial evidence for the importance of Firmicutes in carnivorous birds. The diversity of study designs, species, diet and laboratory procedures used in these studies precludes a meaningful comparison.

### Response of microbiota to captivity

To enable statistical analyses of the sequence data, we focused on a small number of variables; taxonomy (*Accipitriformes/Falconiformes* combined vs. *Strigiformes*), euthanized vs. rehabilitated and time in captivity. Against expectation, host taxonomy was not a significant variable. Despite the anatomical and physiological differences between these orders, this result is consistent with an overall GI anatomy similarity. Possibly, the fact that these birds share a similar diet could be the main factor explaining the similarity in the bacterial taxonomy. The observation that microbiota α-diversity does not appear to be impacted by anti-microbial treatments is of interest. If supported by the analysis of additional samples, this outcome could indicate that intra-muscularly administered antibiotics do not reach a sufficiently high concentration to impact the microbiota. Alternatively, as a result of environmental exposure to antimicrobials, the raptors’ microbiota could be enriched for resistant bacteria, reducing the response of the microbiota to antimicrobials. This interpretation would be consistent with reports of antibiotic resistance in fecal bacteria isolated from wild birds in the Danube delta [29], from sea gulls in Alaska [30] and from other geographical locations [31]. The apparent stability of the fecal microbiota was also apparent when comparing samples from birds that were too sick or injured to be rehabilitated and birds admitted for rehabilitation. This observation could tentatively be explained by the relative short time between injury and euthanasia or death, limiting the extent of changes to the microbiota. The lack of effect of the duration of captivity on the microbiota diversity may indicate that captive raptors rapidly adapted to captivity and did not suffer excessively from stress. It was our expectation to observe decreasing diversity over time, as a result of stress during captivity and consumption of standardized diet.

Although α-diversity was not affected by captivity, we observed statistically significant changes in the microbiota taxonomic profile over time in captivity. The observed increase in *Actinobacteria* in captive birds has to our knowledge not been reported. A search of the literature using various combinations of the terms “raptor”, “carnivore”, “*Actinomycetales*”, “*Actinobacteria*”, “microbiome” and “microbiota” failed to uncover publications specifically discussing the functional importance of this phylum in raptors or in birds, or the impact of captivity on the abundance of this taxon. Some studies have reported a negative correlation between dietary fiber intake and *Actinobacteria* abundance [32], but this view is not universally shared [33]. As discussed above, the paucity of studies of the intestinal microbiome of raptors makes the interpretation of these observation difficult.

## Conclusions

A study of the fecal microbiota of 46 raptors from 7 species was enabled by access to birds admitted to a wildlife rehabilitation facility. In spite of obvious limitations inherent to opportunistic sampling of a heterogeneous population, the approach described here compares favorably with field studies based on trapping or with the study of animals housed for long periods of time in zoos and rehabilitation centers. The large taxonomic distance between fecal microbiotas observed in this study warrants further studies to assess whether this level of heterogeneity is typical of raptors in the wild, of wild animals in general, or was enhanced by the diverse clinical history of the study population.

## Methods

### Birds, medical treatments, diet, sample collection

Birds of prey admitted to TWC (North Grafton, Massachusetts, 42° 14′ 38“ N, 71° 40’ 51” W) due to injury or illness were the subject of this study (Table S2). TWC holds state and federal permits for the rehabilitation of birds of prey. All birds admitted to TWC are examined by wildlife veterinarians, and any diagnostics, such as radiographs, are performed as indicated. Birds for which prognosis for recovery and release back to the wild is determined to be favorable are provided appropriate medical or surgical treatment. Birds for which prognosis for survival and release is determined to be poor are humanely euthanized by induction of general anesthesia with isoflurane gas followed by intravenous injection of pentobarbital sodium and phenytoin sodium.

Medical therapies in individual birds included: antibiotic (ceftiofur crystalline free acid, 20 mg/kg intramuscularly q 4 days); antifungal (itraconazole, 10 mg/kg orally once daily); antiparasitic (praziquantel/pyrantel pamoate/febantel, 5.7–22.7 mg per bird orally once); nonsteroidal anti-inflammatory analgesic (meloxicam, 1 mg/kg orally twice daily or 2 mg/kg orally once daily) and opioid analgesic (long acting buprenorphine, 0.3 mg/kg subcutaneously once daily).

Diets included laboratory-bred mice previously euthanized, frozen and free of drug residues, offered to patients for which the natural diet consists predominantly of mammals. Alternatively, commercially bred quail was offered to patients that naturally consume avian prey. Diets were offered once or twice daily, depending on the need to administer medications in food. *Accipitriformes/Falconiformes* were fed the bulk of their diet, or were fed exclusively, in the morning; *Strigiformes* were fed the bulk of their diet, or were fed exclusively, in the evening.

In live birds, fecal samples were obtained noninvasively by collecting in a plastic syringe case dropping from the floor of the cage or enclosure. As birds were inspected at least twice daily, samples were collect no later than 12 h after defecation. Fecal samples from deceased birds were collected at post-mortem examination by isolating, within 3 h of death, the region of the large intestine from the junction of the ceca to insertion at the cloaca, and extracting intestinal contents into a plastic syringe case. All samples were frozen at − 20 °C until analysis.

The 46 birds from which samples were included in this study are summarized in Table 2. Additional metadata information is found in Table S2.

**Table 2:** Summary of 46 birds included in study

Abbreviation	Species	Common name	Number of birds
AMKE	*Falco sparverius*	American kestrel	1
BAEA	*Haliaeetus leucocephalus*	Bald eagle	2
BDOW	*Strix varia*	Barred owl	8
BWHA	*Buteo platypterus*	Broad-winged hawk	2
COHA	*Accipiter cooperii*	Cooper’s hawk	3
GHOW	*Bubo virginianus*	Great horned owl	1
RTHA	*Buteo jamaicensis*	Red-tailed hawk	29

Of the 46 birds, 4 were considered treated with antibiotics. This designation applies to samples obtained from birds treated with antibiotics for a minimum of 24 h and no more than 5 days after termination of treatment. Bird 9 (w180244) was admitted on 3/28/18, treated from 4/3/18–4/24/18 and sampled on 4/9/18. Bird 12 (w181990) was admitted 7/2/18, treated from 7/3/18–7/15/18 and the sample collected on 7/9/18. Bird 15 (w181930) was admitted on 6/29/18, treated from 6/29/18–7/4/18 and the sample collected 7/9/18. Finally, bird 17 (w181127) was admitted 5/29/18, treated 7/3/18–7/7/18 and the sample collected on 7/9/18 (Table S2).

### Molecular biology and sequencing

#### DNA extraction and PCR procedures

Fecal DNA was extracted in a Qiacube instrument using the Qiagen PowerFecal kit. Two PCRs of 20 temperature cycles each were applied as described [34, 35] to generate amplicons of the bacterial 16S rRNA gene V1V2 variable region [36]. Canonical primers 27F and 338R were used. Uniquely barcoded amplicons were pooled in approximate equal abundance and the multiplexed amplicon library was size-selected on a Pippin HT system (Sage BioScience, Beverly, Massachusetts). The library was sequenced in an Illumina MiSeq sequencer at the Tufts University genomics core facility (tucf.org) using single-end 300-nucleotide chemistry.

#### Analysis of 16S sequence data

Between 27,105 and 120,024 single-end 300-nucleotide sequences were obtained per barcode. Sequences were rarified to 5000 per sample. Sequences were curated using programs found in mothur [37]. Program *screen.seqs* was used to eliminate sequences that did not align, that were unusually short or long, that had ambiguous base calls or homopolymers longer than 8 nucleotides. Curation resulted in the elimination of 15,536 (6.8%) sequences. Sequences were taxonomically classified with program *classify.seqs* using the Silva template and taxonomy files [38]. Sequences were assigned to Operational Taxonomic Units (OTUs) with program *cluster* using the Opticlust method [39]. The distance threshold was set at 3%.

OTU based analyses included 342 OTUs comprising a minimum of 10 sequences. OTUs with fewer than 10 sequences total across the 46 birds were excluded. Subsampling of the 46 samples, for instance when analyzing 22 surviving birds or 24 birds that died or were euthanized, resulted in the exclusion of additional OTUs with no sequence. For instance, of the 342 OTUs included in the entire dataset, 66 were excluded from the analyses of the 22 surviving birds, leaving 276 OTUs.

Two distance metrics were used to compute pairwise distances between microbiota samples. Weighted UniFrac distances [22] were calculated in *mothur*. The EMD distance has been suggested as alternative measure of β-diversity to avoid problems associated with saturation of UniFrac distance when compering highly divergent populations [40]. Because many of the pairwise comparisons gave weighted UniFrac distance values exceeding 0.9 (Fig. S1), the Earth Mover Distance (EMD) was used as an alternative measure of β-diversity [41] to avoid distance saturation problems [40]. EMD values between 1035 pairs (46 × 45/2) of samples were calculated in R with the package Colordistance [42]. Using graphing program Sigmaplot (Systat Software, San Jose, California), OTU profiles for each sample were converted into histograms such that each OTU was assigned a different color. OTUs in the same phylum were assigned similar colors. Histograms were exported in JPEG format and input into the ImageClusterPipeline in Colordistance. The pipeline was run with the following arguments: color.space = “rgb”, distance.method = “emd”, lower = c(0.95,0.95,0.95), upper = c(1.0,1.0,1.0), cluster.method = “kmeans”, kmeans.bins = 69, bin.avg. = TRUE, iter.max = 50. The “upper” and “lower” arguments were applied to remove white pixels in the histograms which have no information. The number of bins was set to 69 because this is the highest number of non-zero OTUs in the 46 samples included in the analyses. Pairwise EMD values were exported in csv format. The Microsoft Excel Add-in GenAlEx [43] was then used to convert distance matrices into Principal Coordinate plots using the PCoA feature in the Distance menu. To assess the effect of selected metadata on the raptors’ microbiota, constrained ordination methods were used. Canonical Correspondence Analysis (CCA) was run in CANOCO [23]. CANOCO uses permutation to assess the significance of the correlation between an independent variable and the dependent variables. In the present case, Z score-normalized OTU abundance values were defined as dependent variables. Avian species and days in captivity were defined as independent variables.

#### Sequence quality control

Sample replication was used to estimate the magnitude of technical variation. Replication consisted in duplicate PCR amplifications and barcoding of randomly selected DNA samples. Measured in EMD units, mean distance between replicates was 0.011 (*n* = 2), equivalent to 2% of the average EMD of 0.527 (*n* = 1035, SD = 0.30) between the 46 raptor microbiota samples. UniFrac distance between replicates averaged 0.077 (*n* = 4, SD = 0.006), which corresponds to approximately 10% of the mean distance of 0.78 for all pairwise distance values (n = 1035, SD = 0.20). The higher relative error obtained with the latter metric is consistent with UniFrac distance saturation.

To control for accuracy of the taxonomic classification of 16S sequences, DNA from a synthetic bacterial population (cat no. HM-782D, BEI Resources, Manassas, Virginia) was amplified using the identical PCR protocol as described above. The amplicon was tagged with a unique 6-nucleotide barcode and included in the 16S library for sequencing. The close match between expected and observed phylum-level classification obtained for this sample is shown in Fig. S4.

## Supplementary information

**Additional file 1: Figure S1**. Rank-abundance plot of weighted UniFrac distance and EMD. UniFrac distance was calculated based on 5000 sequences per sample. EMD was calculated based on the abundance of 342 OTUs. A total of 1035 pairwise distance values were calculated for each distance metric. For clarity, only every 15th data point is shown in each curve. Datapoints are ranked in order of decreasing distance value. Although UniFrac and EMD distance values are significantly correlated (ρ = 0.374 *p* = 2 × 10^− 7^, *n* = 1035), vertically aligned datapoints may not represent the distance between the same pair of samples.

**Additional file 2: Figure S2**. Lin-log plot of microbiota Berger-Parker diversity vs. time in captivity for 46 raptors indicates that captivity does not depress microbiota diversity. Datapoints are colored by species as in Fig. 1. Datapoints at x = 1 represent birds that were euthanized or died within 24 h of admission. Note that the Berger-Parker index is defined as the proportion of the most abundant taxon. Berger-Parker and Shannon diversity therefore tend to be inversely correlated. Color key as shown in Fig. 1.

**Additional file 3: Figure S3**. Intra-muscular injection of antibiotic and oral administration of antifungal does not visibly impact microbiota α-diversity.

**Additional file 4: Figure S4**. Phylum-level classification of 16S sequences from a synthetic bacterial population.

**Additional file 5: Table S1**. Taxonomic classification of 16S sequences from 46 fecal microbiota.

**Additional file 6: Table S2**. Metadata of 46 raptors included in the study.

## Data Availability

The dataset supporting the conclusions of this article is available at the National Center for Biotechnology Information Sequence Read Archive under project accession number PRJEB33803.

## Abbreviations

16S rRNA: Small subunit ribosomal RNA
EMD: Earth mover distance
OTU: Operational taxonomic unit
PCoA: Principal coordinate analysis
CCA: Canonical correspondence analysis
PCR: Polymerase chain reaction
AMKE: *Falco sparverius*
BAEA: *Haliaeetus leucocephalus*
BDOW: *Strix varia*
BWHA: *Buteo platypterus*
COHA: *Accipiter cooperi*
GHOW: *Bubo virginianus*
RTHA: *Buteo jamaicensis*
