# Supplementary figures and images for: The fecal microbiota of wild and captive raptors
Source: Anim Microbiome. 2020 May 6;2:15. doi: 10.1186/s42523-020-00035-7 (PMC7863374; doi:10.1186/s42523-020-00035-7)

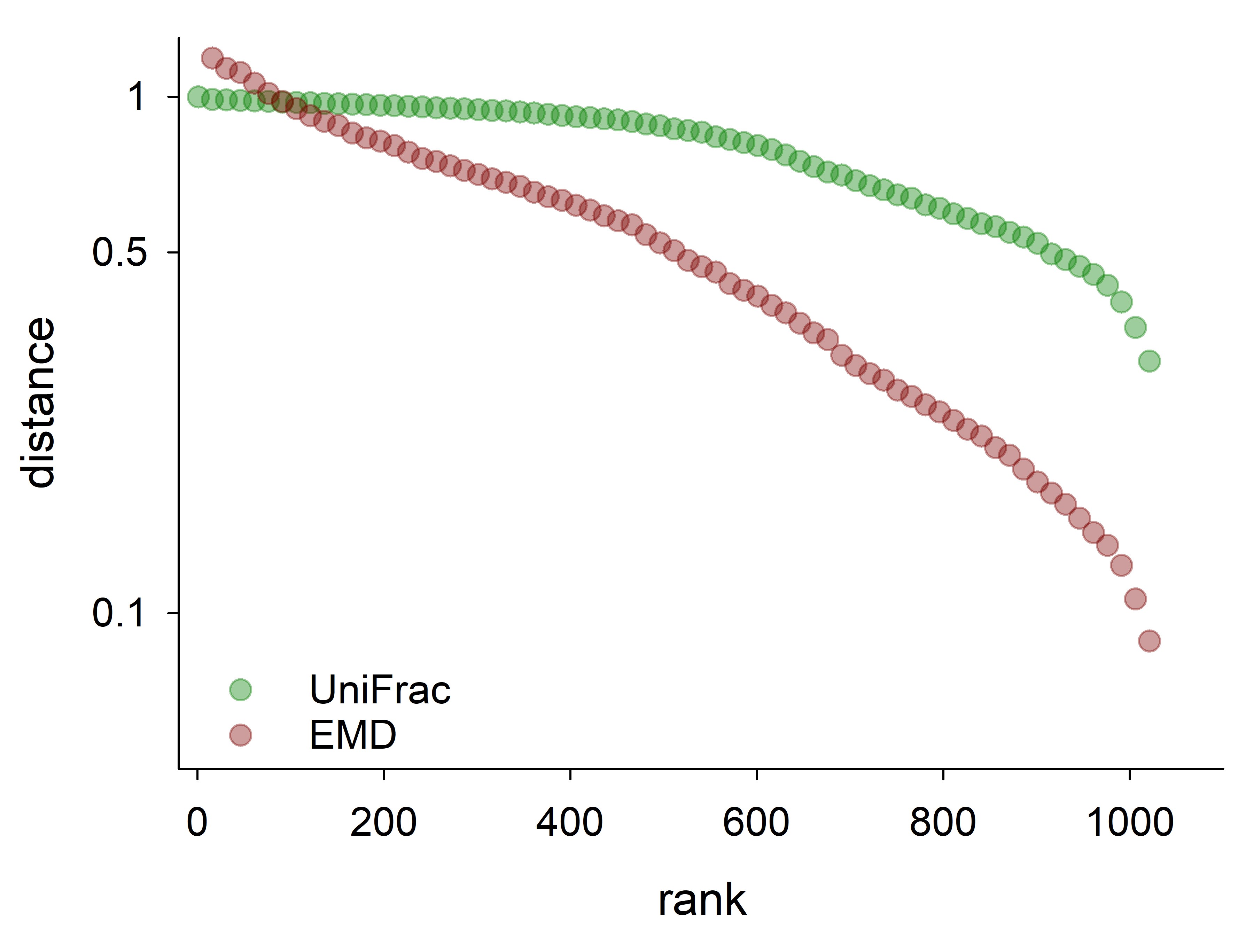

Supplement: Supplementary file 1 — Additional file 1: Figure S1. Rank-abundance plot of weighted UniFrac distance and EMD. UniFrac distance was calculated based on 5000 sequences per sample. EMD was calculated based on the abundance of 342 OTUs. A total of 1035 pairwise distance values were calculated for each distance metric. For clarity, only every 15th data point is shown in each curve. Datapoints are ranked in order of decreasing distance value. Although UniFrac and EMD distance values are significantly correlated (ρ = 0.374 p = 2 × 10− 7, n = 1035), vertically aligned datapoints may not represent the distance between the same pair of samples. [file 42523_2020_35_MOESM1_ESM.jpg]

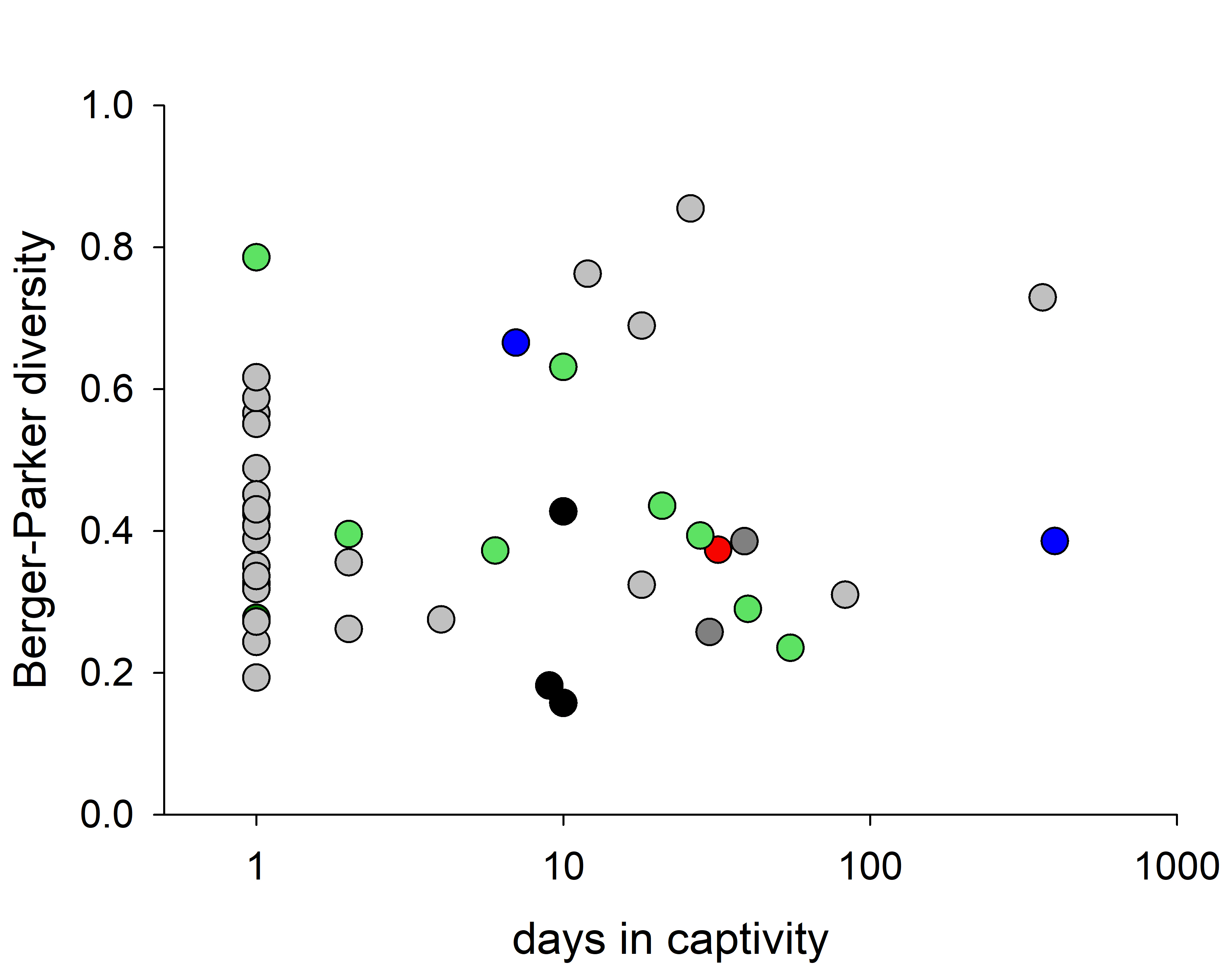

Supplement: Supplementary file 2 — Additional file 2: Figure S2. Lin-log plot of microbiota Berger-Parker diversity vs. time in captivity for 46 raptors indicates that captivity does not depress microbiota diversity. Datapoints are colored by species as in Fig. 1. Datapoints at x = 1 represent birds that were euthanized or died within 24 h of admission. Note that the Berger-Parker index is defined as the proportion of the most abundant taxon. Berger-Parker and Shannon diversity therefore tend to be inversely correlated. Color key as shown in Fig. 1. [file 42523_2020_35_MOESM2_ESM.jpg]

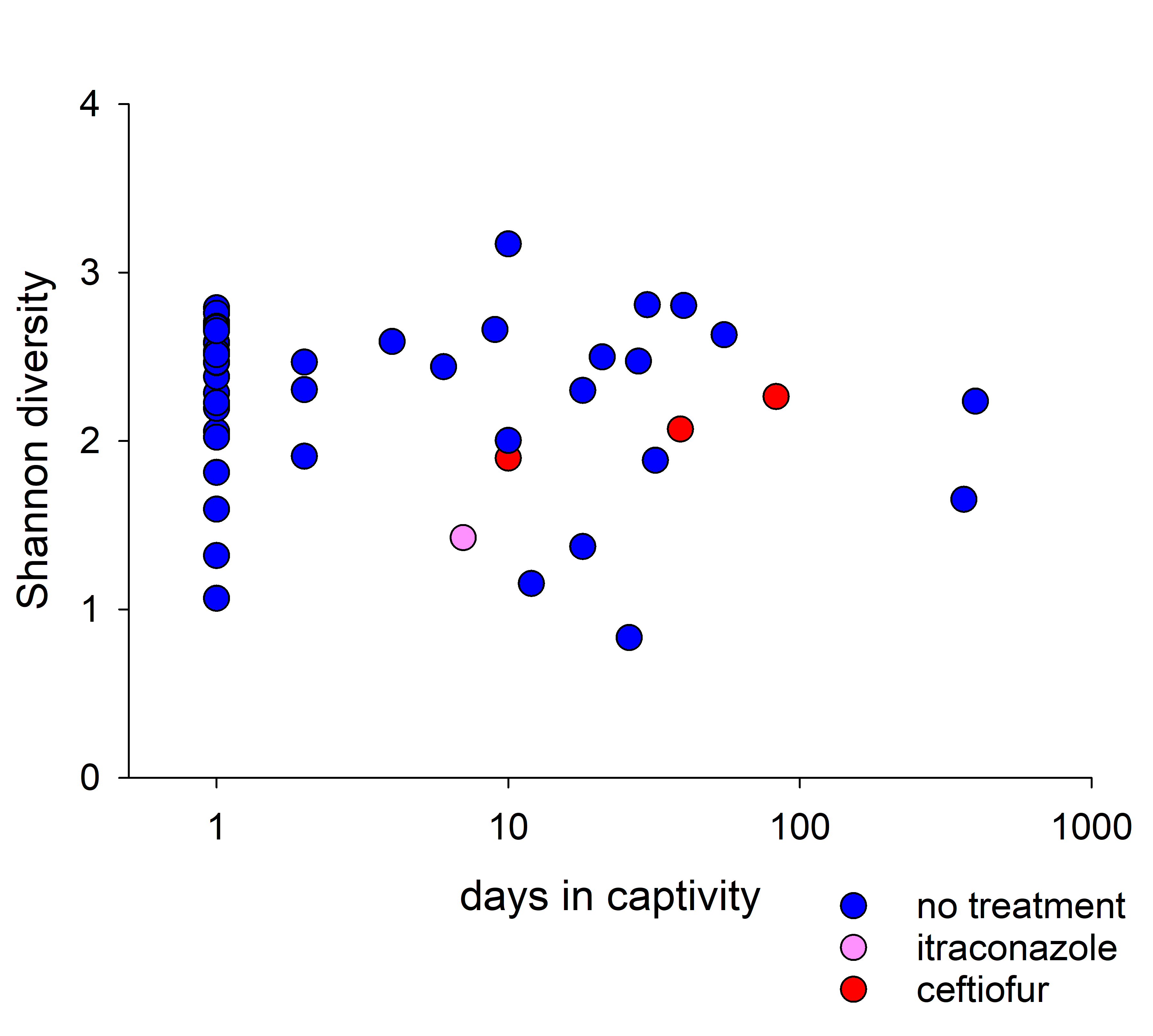

Supplement: Supplementary file 3 — Additional file 3: Figure S3. Intra-muscular injection of antibiotic and oral administration of antifungal does not visibly impact microbiota α-diversity. [file 42523_2020_35_MOESM3_ESM.png]

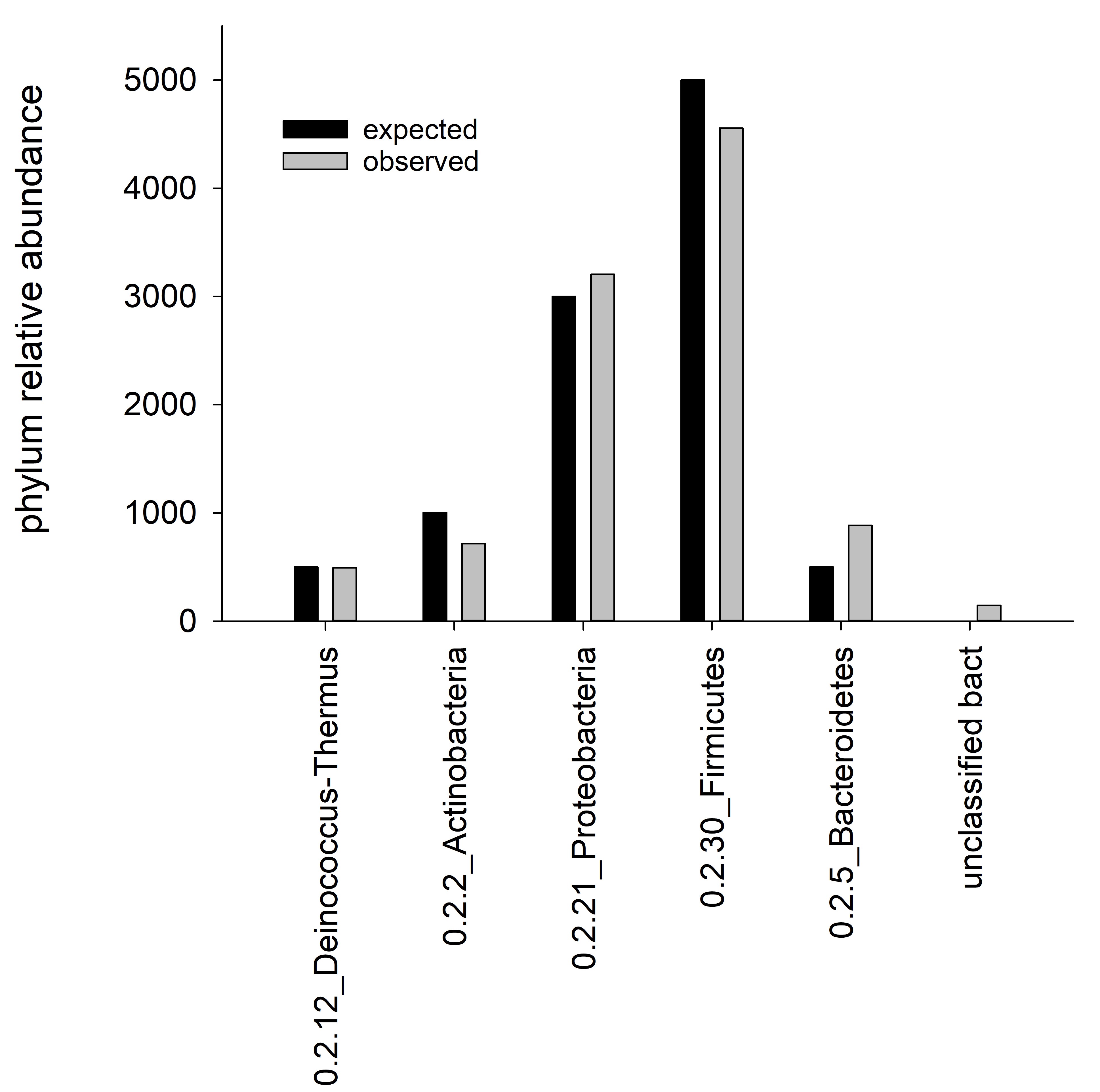

Supplement: Supplementary file 4 — Additional file 4: Figure S4. Phylum-level classification of 16S sequences from a synthetic bacterial population. [file 42523_2020_35_MOESM4_ESM.jpg]
